# Correlations of coronary plaque wall thickness with wall pressure and wall pressure gradient: a representative case study

**DOI:** 10.1186/1475-925X-11-43

**Published:** 2012-07-29

**Authors:** Biyue Liu, Jie Zheng, Richard Bach, Dalin Tang

**Affiliations:** 1 Department of Mathematics, Monmouth University, West Long Branch, NJ, 07764, USA; 2 Mallinckrodt Institute of Radiology, Washington University School of Medicine, St. Louis, MO, 63110, USA; 3 Cardiovascular Division, Washington University School of Medicine, Saint Louis, MO, 63110, USA; 4 Department of Mathematical Sciences, Worcester Polytechnic Institute, Worcester, MA, 01609, USA

**Keywords:** Right coronary artery, Stenosis, Wall thickness, Wall pressure, Pulsatile, Pressure difference, Wall pressure gradient

## Abstract

**Background:**

There are two major hemodynamic stresses imposed at the blood arterial wall interface by flowing blood: the wall shear stress (WSS) acting tangentially to the wall, and the wall pressure (WP) acting normally to the wall. The role of flow wall shear stress in atherosclerosis progression has been under intensive investigation, while the impact of blood pressure on plaque progression has been under-studied.

**Method:**

The correlations of wall thickness (WT) with wall pressure (WP, blood pressure on the lumen wall) and spatial wall pressure gradient (WPG) in a human atherosclerotic right coronary artery were studied. The pulsatile blood flow was simulated using a three dimensional mathematical model. The blood was treated as an incompressible viscous non-Newtonian fluid. The geometry of the artery was re-constructed using an in vivo intravascular ultrasound (IVUS) 44-slice dataset obtained from a patient with consent obtained. The WT, the WP and the WPG were averaged on each slice, respectively, and Pearson correlation analysis was performed on slice averaged base. Each slice was then divided into 8 segments and averaged vessel WT, WP and WPG were collected from all 352 segments for correlation analysis. Each slice was also divided into 2 segments (inner semi-wall of bend and outer semi-wall of bend) and the correlation analysis was performed on the 88 segments.

**Results:**

Under mean pressure, the Pearson coefficient for correlation between WT and WP was r = − 0.52 (p < 0.0001) by 2-segment analysis and r = − 0.81 (p < 0.0001) by slice averaged analysis, respectively. The Pearson coefficient for correlation between WT and WPG was r = 0.30 (p = 0.004) by 2-segment analysis and r = 0.45 (p = 0.002) by slice averaged analysis, respectively. The r-values corresponding to systole and diastole pressure conditions were similar.

**Conclusions:**

Results from this representative case report indicated that plaque wall thickness correlated negatively with wall pressure (r = −0.81 by slice) and positively with wall pressure gradient (r = 0.45). The slice averaged WT has a strong linear relationship with the slice averaged WP. Large-scale patient studies are needed to further confirm our findings.

## Introduction

It is believed that mechanical stresses are associated with the pathogenesis of atherosclerosis in the human arterial tree [1,2]. The luminal surface of the blood vessel and its endothelia surface are constantly exposed to hemodynamic wall shear stress (WSS) and wall pressure (WP). The role of flow WSS in atherosclerosis progression and the correlation of the low WSS with the wall thickness (WT) of stenotic coronary arteries have been studied in great detail [3-6]. It is well known that both low WSS and high oscillatory patterns of WSS cause intimal wall thickening [3-6]. There is also strong clinical evidence suggesting that blood pressure is a major determinant of vascular changes in the arterial system. Thubrikar and Salzar presented the hypothesis that arterial wall stress and accompanying stretch, produced by intraliminal pressure, are major contributing factors to the localization of atherosclerotic lesions [7,8]. Elevated blood pressure is closely associated with symptomatic atherosclerotic disease and localization of plaques [1]. Leary [9] and Willis [10] have suggested that the frequent selective localization of atherosclerotic plaques in coronary arteries is related to the increased blood pressure in the coronary arteries.

During the last few decades, extensive research work has been done on the role of hypertension in coronary heart disease [11-16]. Kannel et. al. [14] have explored prospectively in some detail the relation of antecedent blood pressure status to the risk of subsequent clinical manifestations of coronary heart disease over 14 years in a cohort of 5127 men and women in Framingham, Massachusetts. In his review, Chobanian [12,15] examined the influence of hypertension on the development of atherosclerosis and of cardiovascular complications in man and experimental animals. An association between hypertension and coronary heart disease is well established. Obstruction to blood flow is accompanied by a pressure drop across the stenosis (ΔP = P_e_ – P_a_, where P_e_ and P_a_ are the normal aortic pressure and the pressure distal to the stenosis, respectively). Extensive studies suggest that the pressure drop across a single discrete coronary stenosis is given by a stenosis pressure drop-flow relationship having the general form ΔP = AQ + BQ^2^[17-21], where Q is the flow rate or velocity; coefficients A and B depend on stenosis morphology and rheological properties of blood, including the normal diameter or cross-section area of the artery and the minimum diameter or cross-section area of stenosis. The magnitude of the pressure drop has been clinically used to judge severity of the lesion, and the reduction in the pressure drop due to angioplasty is used to judge the success of an interventional procedure. Anderson et. al. [17] examined 4263 sets of measurements in patients who underwent percutaneous transluminal coronary angioplasty on single, discrete coronary artery lesions. Their study suggested that the measurement of the pressure drop can be a useful indicator of the final diameter stenosis measurement and the magnitude of the reduction of diameter stenosis.

Even though the relationship between individual blood pressure level and the atherosclerotic disease in coronary arteries has been extensively investigated during the past decades and it is now acknowledged that a pressure drop is associated with the stenosis, little work has been reported on the relationship of local blood pressure with the right coronary plaque. Despite the fact that the regional flow measurement techniques have been progressed significantly it is still very difficult to make detailed pressure and flow measurement to describe in detail the flow mechanism locally in atherosclerotic coronary arteries. Computational fluid dynamics has been proven to be a reliable tool to gain localized information into the complex blood flow fields in coronary arteries. Giannoglou et. al. [22] used the models of two-dimensional steady flow to examine the correlation between the development of vessel wall thickening and the blood flow physical parameters. They found that the low static pressure was significantly correlated with increased vessel wall thickening.

In the present work we investigated the blood flow in atherosclerotic right coronary artery using an unsteady three-dimensional model based on patient-specific plaque geometry. The objectives of this study were to quantitatively examine the correlations of the vessel wall thickness (WT) with the blood pressure (locally), such as the pressure difference, the wall pressure (WP, blood pressure on the lumen wall) and the spatial wall pressure gradient (WPG), and to examine the spatial and temporal patterns of the blood pressure in stenotic human right coronary arteries.

## Methods

### Patient-specific plaque geometry based on IVUS data

**Figure 1:**
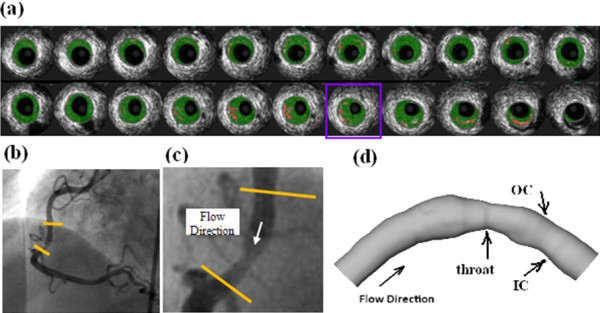
(a) Selected IVUS slices from a 44-slice set; (b) Angiographic image showing location of the imaged coronary segment (c) Enlarged view of the segment and flow direction (d) Geometry of the computational domain.

**Figure 2:**
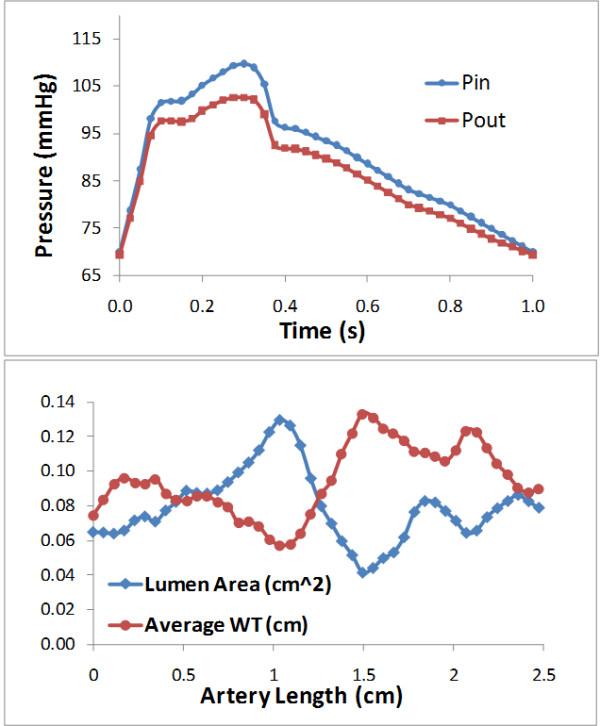
**Top: Pulsatile pressure waveform at the inlet and the outlet. Bottom: Lumen cross sectional area and the average WT from the inlet to the outlet.** Throat of the stenosis at s = 1.49.

The arterial geometry plays an important role in determining the localized blood flow information. Thus hemodynamic studies based on patient specific data are likely to offer insights on its role in atherogenesis. An image-based model of an atherosclerotic right coronary artery was re-constructed based on the lumen contour curves extracted from a 44-slice in vivo 3D IVUS dataset covering the plaque region, which was acquired during cardiac catheterization from a patient, using a 20-MHz, 2.9-F phased-array Eagle Eye Gold IVUS catheter (Volcano Corporation, Rancho Cordova, CA). Figure. 1 (a) presents selected 22 slices from the 44-slice IVUS dataset. The slice enclosed in a purple box was the IVUS slice at the throat of stenosis. The single plane X-ray angiographic data (Figure. 1 (b)) was used for the determination of the curvature to align IVUS slices. The contour plots of lumen and vessel out-boundary were automatically generated by an in-house software atherosclerotic plaque imaging analysis (APIA) written in MATLAB. The distance from each point on the lumen boundary to the vessel out-boundary was calculated and counted as the wall thickness. Extensive details of the processing of the images can be found in reference [23]. The lumen cross sectional area from the inlet to the outlet was plotted in Figure. 2 (bottom), where the horizontal axis is the artery length with the inlet at s = 0 and the outlet at s = 2.47 *cm*. The minimum lumen cross sectional area at the throat of stenosis is 0.042 *cm*^*2*^. The maximum area of the cross section proximal to the stenosis is 0.130 *cm*^*2*^. The reduction of the lumen cross sectional area at the throat of stenosis is approximately 68 %.

### The flow model

The blood was assumed as a laminar, incompressible, non-Newtonian viscous fluid. The time dependent three dimensional Mass-Momentum equations were used as the governing equations. A no-slip condition was applied to the velocities at the lumen wall, treated to be inelastic and impermeable. A time dependent pressure with waveform P_In_ and P_Out_ (as shown in Figure. 2 (top)) was imposed at the inlet and outlet boundary, respectively. This pressure waveform was from another patient. Its shape represents a typical pressure waveform in coronary artery. The pressure was assumed to be uniform over the cross section of the inlet and the outlet. The initial conditions for the velocity and the pressure were obtained by solving the system of steady state Mass-Momentum equations. The calculated flow velocity at the inlet is consistent with the averaged inlet velocity values for right coronary arteries listed in Table 1 in [22]. The blood was treated as a non-Newtonian fluid obeying the Carreau model with the viscosity-shear rate relation:

(1) $$\eta =\eta_{\infty }+(\eta_{0}-\eta_{\infty }){\left[1+{\left(\lambda \dot{\gamma }\right)}^{2}\right]}^{\frac{n-1}{2}}$$

where η_0_ = 0.056 *Pa·s* is the zero shear rate viscosity, $\eta_{\infty }$= 0.00345 *Pa·s* is the infinite shear rate viscosity, λ = 3.313 *s* is a parameter, and n = 0.3568 is a dimensionless parameter [24,25]. In the computations, the blood density ρ was assumed to be constant at 1050 *kg*/*m*^*3*^.

### Solution method

The finite element method over a tetrahedral mesh was adopted to solve the governing equations of the fluid motion into the right coronary artery. Computations were performed using COMSOL 4.2. The inlet and outlet were extended in length by .2 cm in the direction normal to the inlet and outlet cross sections to reduce the influence of the artificial boundary conditions in the region of interest. Four consecutive cardiac cycles were simulated to ensure that the flow was truly periodic. To confirm the independence of the numerical solutions on spatial mesh, computations were repeated over the following three meshes: a mesh of 67264 elements with the number of degrees of freedom as 169660, a mesh of 148990 elements with the number of degrees of freedom as 356544, and a mesh of 208405 elements with the number of degrees of freedom as 493544. The relative errors of the solutions between different meshes were less than 0.5 %. The numerical results presented here were obtained based on an unstructured finite element mesh containing 148990 elements.

### Notations and the approach in data analysis

The pressure difference along the artery length is defined as P-P_In_, where P_In_ is a reference pressure chosen simultaneously as the blood pressure at the inlet of the coronary artery. The WPG is defined as

(2) $$WPG=\sqrt{{\left(\frac{\partial p}{\partial x}\right)}^{2}+{\left(\frac{\partial p}{\partial y}\right)}^{2}+{\left(\frac{\partial p}{\partial z}\right)}^{2}}$$

which is the spatial gradient of the wall pressure. Since the artery is asymmetric, there is no exact centre plane of curvature of the bend. The axial cross section of the artery with x = 0 would serve approximately as the centre curvature plane. This centre curvature plane intersects the lumen boundary with two curves: one is on the outer border of the bend which will be referred as the outer curve (OC) in later discussions; the other is on the inner border of the bend referred as the inner curve (IC).

The artery was sectioned by 44 cross-sectional slices along the artery length from the inlet (slice #1) to the outlet (slice #44) with the throat of the stenosis at the slice # 27 (selected 22 slices were shown in Figure. 1(a)). The lumen and wall contours for each cross-section were used to determine the local vessel wall thickness at locations corresponding to the lumen mesh points. In the quantitatively statistic analysis, 96 mesh points were picked approximately evenly spaced on the lumen contour of each slice. The WT, the WP and the WPG were averaged on the picked mesh points of each slice, respectively, and Pearson correlation analysis was performed on slice averaged base. Each slice was then divided into 8 segments, and averaged vessel WT, WP and WPG were collected from all 352 segments to perform 8-segment based correlation analysis. Each slice was also divided into 2 segments (inner semi-wall of bend and outer semi-wall of bend) and the correlation analysis was performed on the 88 segments.

## Results

### Correlations of WT with WP and WPG

**Figure 3:**
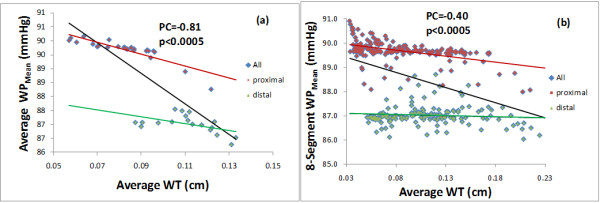
**Scatter plots of (a) the slice averaged WT vs. the slice averaged WP and (b) the WT vs. the WP by 8-segment analysis, under mean pressure.** The Pearson coefficients for the correlation between WT and WP are r = −0.81 by slice and r = −0.40 by 8-segment analysis, respectively, with *p-values* < 0.0001.

Tables 1 and 2 present the Pearson correlations of the WT with the WP and the WPG, respectively, based on the analyses by segment and by slice at systole (t = 0.05) and at the end of diastole (t =1). Also the time averaged mean in a cardiac cycle of the WP and WPG were calculated at each point on the lumen surface, and the correlations of the WT with the WP_Mean_ and WPG_Mean_ were included in Tables 1 and 2. Under mean pressure, the Pearson coefficients for correlation between the WT and the WP were r = − 0.40 (p < 0.0001) by 8-segment analysis, r = − 0.52 (p < 0.0001) by 2-segment analysis, and r = − 0.81 (p < 0.0001) by slice averaged analysis, respectively. The Pearson coefficients for correlation between WT and WPG were r = 0.24 (p < 0.0001) by 8-segment analysis, r = 0.30 (p = 0.004) by 2-segment analysis, and r = 0.45 (p = 0.002) by slice averaged analysis, corresponding to mean blood pressure. The r-values corresponding to systole and diastole pressure conditions were similar. Figure. 3 presents the scatter plots with trend lines of (a) the slice averaged WT vs. the slice averaged WP and (b) the WT vs. the WP by segment (each slice was divided by 8 segments) under mean pressure. The Pearson correlation analysis revealed strong correlations of the slice averaged WT with the slice averaged WP under systolic, diastolic and mean blood pressure. The plots in Figure. 3 also show two clusters which represent different segments of the artery: one contains the data of the slices from inlet to the neck of stenosis (proximal) and the other contains the data of the rest slices (distal). The trend line for the overall data is in black. The trend line for proximal and distal cluster data is in red and in green, respectively. In Figure. 3(a) by slice averaged analysis both clusters have trend lines with strong negative correlations between average WT and average WP_mean._ In Figure. 3(b) the trend line for distal cluster has a very small negative slope, while the trend line for the overall data clearly has a negative slope. This may suggest that the overall negative correlation between the WT and the WP_mean_ by segment is mainly due to the pressure drop across the stenosis.

**Table 1:** Correlation of WT with WP, based on the analyses by segment and by slice at systole (t = 0.05), the end of diastole (t =1), and the time averaged mean in a cardiac cycle

**t**	**By segment (8 segments/slice)**		**By segment (2 segments/slice)**		**By slice averaged**	
	**P Coef.**	** *p* -Value**	**P Coef.**	** *p* -Value**	**P Coef.**	** *p* -Value**
Mean	−0.40	<0.0001	−0.52	<0.0001	−0.81	<0.0001
t = 0.05	−0.35	<0.0001	−0.45	<0.0001	−0.71	<0.0001
t = 1	−0.41	<0.0001	−0.54	<0.0001	−0.84	<0.0001

**Table 2:** Correlation of WT with WPG, based on the analyses by segment and by slice at systole (t = 0.05), the end of diastole (t =1), and the time averaged mean in a cardiac cycle

**t**	**By segment (8 segments/slice)**		**By segment (2 segments/slice)**		**By slice averaged**	
	**P Coef.**	** *p* -Value**	**P Coef.**	** *p* -Value**	**P Coef.**	** *p* -Value**
Mean	0.24	<0.0001	0.30	0.004	0.45	0.002
t = 0.05	0.25	<0.0001	0.32	0.003	0.43	0.003
t = 1	0.23	<0.0001	0.29	0.004	0.45	0.002

Table 3 presents the correlations of the lumen cross-section area (LA) with the slice averaged WP and WPG at systole (t = 0.05) and at the end of diastole (t =1). Also the time averaged mean in a cardiac cycle of the WP and WPG were calculated on the lumen surface, and the correlations of the LA with the WP_mean_ and WPG_mean_ were included. Under mean pressure, the Pearson coefficients for correlation of the LA with the slice averaged WP and WPG were r = 0.56 (p < 0.0001) and r = −0.41 (p = 0.008), respectively. The r-values corresponding to systole and diastole pressure conditions were similar.

**Table 3:** Correlation of LA with slice averaged WP and WPG at systole (t = 0.05), the end of diastole (t =1), and the time averaged mean in a cardiac cycle

**t**	**LA vs. WP**		**LA vs. WPG**	
	**P Coef.**	** *p* -Value**	**P Coef.**	** *p* -Value**
Mean	0.56	<0.0001	−0.41	0.008
t = 0.05	0.40	0.007	−0.44	0.003
t = 1	0.62	<0.0001	−0.40	0.008

### Local flow patterns and the hemodynamic environment

**Figure 4:**
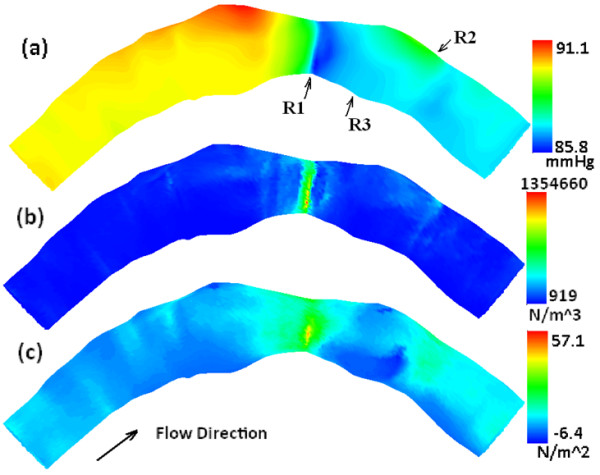
Spatial distribution of the time averaged mean in a cardiac cycle of (a) WP, (b) WPG, and (c) WSS along the artery.

**Figure 5:**
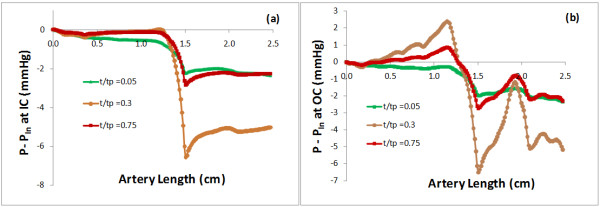
**Pressure difference (P-P**_**In**_**) at various instants of time during the cardiac cycle (a) along the inner boundary line (IC) and (b) along the outer boundary line (OC)**.

To examine the local flow pattern and the hemodynamic environment, the wall shear stress (WSS) was also calculated. Figure. 4 presents the spatial distributions of the time averaged mean in a cardiac cycle of (a) the WP, (b) the WPG, and (c) the WSS along the artery, respectively. Figure. 5 plots the pressure difference P-P_In_ (a) along the inner curve (IC) and (b) along the outer curve (OC) at various instants of time during the cardiac cycle: beginning of systole (t =0.05), peak of systole (t =0.30), and middle of diastole (t =0.75). The horizontal axis is the artery length with the inlet at s = 0, the outlet at s = 2.47 *cm*, and the throat of the stenosis at s = 1.49 *cm*. At the inlet the calculated mean and peak axial velocities were 0.53 m/s and 0.76 m/s, respectively. It is consistent with the averaged inlet velocity values for right coronary arteries listed in Table 1 in [22]. The mean and peak flow rates were 3.38 cm^3^/s and 4.85 cm^3^/s, respectively. The mean and peak Reynolds numbers were 392 and 581, respectively. The mean and peak Dean numbers were 138 and 204, respectively. Womersley number was 1.82.

Plots in Figures 4 and 5 show that the WP, the WPG and the WSS are highly non-uniformly distributed on the lumen wall of the atherosclerotic right coronary artery. Comparing the spatial distribution patterns of three contours in Figure 4, we can note the following patterns on three areas of interest: 1) the area with the global minimum of the WP, the global maximum of the spatial WPG and the global maximum of the WSS at the throat of the stenosis, where the wall thickness assumes the maximum (marked as R1 on Figure 4 (a)); 2) the area with locally high WP (recovered), locally high WPG and locally high WSS on the outer bend of the distal region (marked as R2); and 3) the area with relatively low WP, low WPG and low WSS on the inner bend of the distal region (marked as R3). Plots in Figure 5 can further confirm above observations quantitatively. From Figures 4 and 5 we can also see that the WP behaviors differently along the inner bend from along the outer bend. The WP stays stable at a relatively low level along the IC (see Figure 5 (a)), which indicates that after recovering slightly from a falling to the minimum at the throat of the stenosis, the pressure in distal region and downstream stays constantly low on the inner side of the bend in the stenotic right coronary artery. The peak of the WP in the proximal region and the oscillations of the WP in downstream along the OC (see Figure 5 (b) and Figure 4 (a)) might have been caused by the expansions of lumen area in the regions. The plots in bottom panel of Figure 2 clearly show that each region with an elevation of the WP along the outer bend in Figures 4 and 5 is associated with a relatively small value of the WT and a relatively large value of lumen area.

## Discussions

There are two major hemodynamic stresses imposed at the blood-arterial wall interface by flowing blood. One is the WSS acting tangentially to the wall, and another is the WP acting normally to the wall. These forces influence the artery wall metabolism and correspond to the local modifications of artery wall thickness, composition, microarchitecture, and compliance. While previous studies have focused largely on the correlation between the low WSS and the luminal diameter of coronary arteries [2,6], this study investigates the correlation between the WT of a stenotic right coronary artery with the local blood pressure. The blood pressure difference along the coronary arterial length and the local magnitude of the spatial WPG are believed to be important initiating factors for atherosclerosis and intimal hyperplasia development. Our observation on the correlation of the WT with local WP is in agreement with a reported result obtained using two dimensional models [22].

The change in WT without a formation of focal lesion might not cause an alteration of WP and WPG. The lumen geometry will be changed after the formation of focal lesion, resulting in the change in the patterns of the local WP and WPG on the lumen surface in stenotic region and downstream. Figure 4 demonstrates that the WP, the WPG and the WSS all vary markedly across the stenosis. In the distal region and downstream, the area on the inner side of bend experiences constantly low WP in a cardiac cycle, while the area on the outer side of bend experiences a local elevation in the WP. Blood pressure is a dominant contributor to the major cardiovascular diseases, particularly for stroke and cardiac failure [14]. The atherosclerotic materials probably move within the sub-endothelial layer into regions of low WP. Along the vessel, at regions where low WSS and locally low WP occur, the effect of blood flow resistance, due to increased blood molecular viscosity, gives rise to increased contact time between the atherogenic particles of the blood and the endothelium [26,27]. The components of the WPG possibly have different effects upon endothelial cells. The WPG as well as the WSS gradient in space and in the phase of the cardiac cycle may represent important local modulators of endothelial gene expression in atherogenesis [27,28]. The WPG acting on the endothelium gives rise to a torque development resulting in the redistribution of the initially accumulated atheromatic material within the sub-endothelial layer [26]. Blood pressure elevation will generally induce a variety of changes in the arterial wall. Its potential effect on atherogenesis has been attributed to increased arterial wall permeability, mechanical modification of smooth muscle metabolism, and to increased cell proliferation and matrix deposition [29]. Focal intimal thickening is a consistent feature of prolonged blood pressure elevation and results from accumulation of cellular as well as extracellular constituents [12].

Tables 1 and 2 show that the Pearson correlation analysis by slice reveals a stronger linear relationship between the WT and the WP than that by segment in this case. It is well known that the local blood pressure distribution can be affected by geometric factors, such as the curvature of bend, location and size of stenosis, etc. [26,27,30]. Among these factors, the change of the lumen cross sectional area plays an important role on the variation of the WP. From the selected IVUS slices shown in Figure 1(a) we can see that the distance from the lumen contour to the wall contour is not uniform on a cross-section of the narrowed artery. Thus the cross sectional lumen area of a slice can be better reflected by the slice averaged WT than by the WT of segment. This can probably explain why the Pearson correlation analysis by slice reveals stronger linear relationships of the WT with the WP and the spatial WPG than those by segment analysis. Another possible reason could be that the geometry of the artery was constructed with single-plane angiogram when aligning IVUS slices. The calculated wall pressure may not sensitively respond to the change of the WT if the maximum wall thickness of a cross-section occurs on the sides of the bend. Thus, the segment correlation could be weakened while the slice averaged correlation might not be significantly affected.

The total pressure drop across a stenosis is the sum of viscous losses due to friction and losses incurred at the exit after acceleration along the throat of the lesion [21]. The pressure drop-flow relationship [17-21] can be used to calculate the total pressure drop across the stenosis and to assess the physiological significance of a stenosis on coronary blood flow. However, it does not provide the information of the detailed local pressure distribution (both spatial and temporal) along the lumen surface in stenotic region. Such information could be very helpful in understanding the local hemodynamic environment which constitutes a major determinant of focal atherogenesis and atherosclerotic plaque development [31,32]. The correlation analysis presented in this work examines the local WP and WT quantitatively in stenotic region of right coronary arteries. A comparison of the first column in Table 3 with the third column in Table 1 may also suggest that the slice averaged WP correlates to the slice averaged WT with a stronger linear relationship than that to the lumen cross-section area (LA).

### Limitations

We would like to acknowledge the following limitations: a). Since the image data and boundary conditions are from different patients, the results presented here needs to be further evaluated for a patient-specific case, comprising of image data and boundary conditions from the same patient; b). The vessel curvature was based on a single plane (2D) angiographic data which is only an approximation to the actual 3D curvature; c). Plaque material properties from the current literature were used since patient-specific data was not available; d). The local WP based on the blood flow simulation in the artery is a function of the lumen geometry. The analysis might not be able to reveal strong correlations of the WT with WP and WPG in the situation in which WT variation is notable, but there is not yet a stenosis that affects the lumen.

## Conclusion

This study investigated the correlations of the WT with the local WP and the WPG and the blood pressure patterns in a patient specific right coronary artery. Results from this representative case report indicated that plaque wall thickness correlated negatively with wall pressure (r = −0.81 by slice) and positively with wall pressure gradient (r = 0.45). The slice averaged WT has a strong linear relationship with the slice averaged WP in this case. The blood pressure falls rapidly in the stenotic region, reaches the minimum at the throat of the stenosis, and recovers partially in the distal region and downstream. For stenotic coronary arteries with different size of stenosis and different length of artery, the values of the correlation coefficients may vary. Large-scale patient studies are needed to further confirm our findings.

## Competing interests

Other than the grants listed in the acknowledgement section, the authors declare that they have no other competing interest.

## Authors’ contributions

All authors actively contributed to the research and the writing of the manuscript. BL and DT contributed to the computational modeling, data analysis, and the draft of the manuscript. JZ and RB contributed to the image acquisition, processing and data analysis, and writing of the manuscript. All authors have read and approved the final manuscript.
